# Baseline Plasma C-Reactive Protein Concentrations and Motor Prognosis in Parkinson Disease

**DOI:** 10.1371/journal.pone.0136722

**Published:** 2015-08-26

**Authors:** Atsushi Umemura, Tomoko Oeda, Kenji Yamamoto, Satoshi Tomita, Masayuki Kohsaka, Kwiyoung Park, Hiroshi Sugiyama, Hideyuki Sawada

**Affiliations:** Department of Neurology and Clinical Research Center, National Hospital of Utano, National Hospital Organization, Kyoto, Japan; Chiba University Center for Forensic Mental Health, JAPAN

## Abstract

**Background:**

C-reactive protein (CRP), a blood inflammatory biomarker, is associated with the development of Alzheimer disease. In animal models of Parkinson disease (PD), systemic inflammatory stimuli can promote neuroinflammation and accelerate dopaminergic neurodegeneration. However, the association between long-term systemic inflammations and neurodegeneration has not been assessed in PD patients.

**Objective:**

To investigate the longitudinal effects of baseline CRP concentrations on motor prognosis in PD.

**Design, Setting, and Participants:**

Retrospective analysis of 375 patients (mean age, 69.3 years; mean PD duration, 6.6 years). Plasma concentrations of high-sensitivity CRP were measured in the absence of infections, and the Unified Parkinson’s Disease Rating Scale Part III (UPDRS-III) scores were measured at five follow-up intervals (Days 1–90, 91–270, 271–450, 451–630, and 631–900).

**Main Outcome Measure:**

Change of UPDRS-III scores from baseline to each of the five follow-up periods.

**Results:**

Change in UPDRS-III scores was significantly greater in PD patients with CRP concentrations ≥0.7 mg/L than in those with CRP concentrations <0.7 mg/L, as determined by a generalized estimation equation model (*P* = 0.021) for the entire follow-up period and by a generalized regression model (*P* = 0.030) for the last follow-up interval (Days 631–900). The regression coefficients of baseline CRP for the two periods were 1.41 (95% confidence interval [CI] 0.21–2.61) and 2.62 (95% CI 0.25–4.98), respectively, after adjusting for sex, age, baseline UPDRS-III score, dementia, and incremental L-dopa equivalent dose.

**Conclusion:**

Baseline plasma CRP levels were associated with motor deterioration and predicted motor prognosis in patients with PD. These associations were independent of sex, age, PD severity, dementia, and anti-Parkinsonian agents, suggesting that subclinical systemic inflammations could accelerate neurodegeneration in PD.

## Introduction

Parkinson disease (PD), a common neurodegenerative disorder mainly affecting elderly individuals, is characterized by bradykinesia, muscular rigidity, and tremor, which are caused by progressive dopaminergic neuronal degeneration. PD is pathologically characterized by the presence of Lewy bodies, which consist mainly of aggregated α-synuclein. Although the pathogenesis of this disease has not been completely elucidated, inflammatory and cell death pathways are altered in the brain and peripheral blood of PD patients [1]. Additionally, activated microglial cells have been identified in the substantia nigra of the brain in these patients [2,3]. Aggregated α-synuclein released from neurons in PD can stimulate the secretion of inflammatory cytokines, which activate microglia and inflammasomes and induce neuroinflammation [4]. In animal models of PD, systemic inflammatory stimuli can promote neuroinflammation by accelerating dopaminergic neurodegeneration in the substantia nigra [5–8]. Although we previously demonstrated that acute systemic inflammation can worsen PD motor symptoms [9], the association between chronic systemic inflammations and neurodegeneration in PD patients has not yet been investigated.

C-reactive protein (CRP), a biomarker of systemic inflammation in peripheral blood, has been associated with the development of Alzheimer disease [10,11]. Elevated CRP increases the permeability of the blood–brain barrier by binding to the Fcγ receptor [12], resulting in the activation of microglia in the brain [13]. Affected brain areas in patients with Alzheimer disease have been found to contain both activated microglia and CRP [14,15]. CRP is synthesized in hepatocytes in response to activation by inflammatory cytokines [16,17]. Although plasma CRP concentrations are elevated transiently and dramatically during acute inflammation [18], they are stable in the absence of inflammation [19]. Thus, CRP concentrations in clinically non-inflammatory conditions may affect long-time prognosis by enhancing the neurodegenerative process.

This study investigated the association between CRP concentrations and functional motor deterioration in patients with PD using the Unified Parkinson’s Disease Rating Scale Part III (UPDRS-III) score [20,21]. The hypothesis, that high plasma CRP concentration would enhance the neurodegenerative process and accelerate functional deterioration in patients with PD, was tested.

## Materials and Methods

### Study Design

This retrospective cohort study investigated the association between baseline CRP concentration and motor function in patients with PD. As the primary outcome measure, we evaluated the change in UPDRS-III score from study enrollment to each of five follow-up periods (Days 1–90, 91–270, 271–450, 451–630, and 631–900). The association between baseline CRP and change in UPDRS-III score was evaluated as the regression coefficient of baseline CRP that was calculated using a generalized estimation equation model for the entire follow-up period (Analysis I), and a generalized linear model for the final follow-up period (Days 631–900; Analysis II).

### Ethical approval

This study was approved by the Bioethics Committee of Utano National Hospital, which waived the requirement for informed consent because of to the retrospective nature of the study and the anonymous data analysis. The study protocol was consistent with the principles of the Declaration of Helsinki and the Ethical Guidelines for Medical and Health Research Involving Human Subjects of the Ministry of Health, Labour and Welfare, Japan.

### Study Subjects

#### PD patients

All PD patients fulfilled the United Kingdom Parkinson’s Disease Society Brain Bank clinical diagnostic criteria (steps 1 and 2) [22] and were admitted to the Department of Neurology of the National Regional Center for Neurological Disorders and National Hospital of Utano between January 2008 and December 2013. Subjects were included in the study if (i) they did not have infections when their plasma concentrations of high-sensitivity CRP were measured, and (ii) they had modified Hoehn and Yahr (H-Y) stage 4 or less at baseline. Subjects were excluded if (i) they developed dementia within 1 year of the onset of parkinsonism, based on consideration of dementia with Lewy bodies (1-year rule) [23]; (ii) they had a history of brain surgery; (iii) they had any infection within one month prior to study enrollment; (iv) they had any concomitant chronic inflammatory disease including autoimmune disease or malignancy; or (v) they were taking corticosteroids at study enrollment.

#### Controls

Subjects (matched by age and sex to PD patients) who were admitted to the Department of Cardiology, Gastroenterology, Respiratory disorders or the Department of Metabolomics of the National Hospital of Utano and who met the abovementioned criteria were enrolled as controls. Patients diagnosed with parkinsonism were excluded from this group.

### Evaluation of extrapyramidal signs, inflammatory molecules, and other characteristics

Parameters measured in all patients included modified H-Y stage and UPDRS-III scores for extrapyramidal signs, as well as plasma concentrations of high-sensitivity CRP. In PD patients with “on-off” or “wearing-off” phenomena, modified H-Y stage and UPDRS-III scores were determined during an “on period”. Data on daily doses of L-dopa, entacapone, dopamine agonists, selegiline, and amantadine, and the use of anti-psychotic drugs, were recorded. L-dopa equivalent doses (LEDs) for each of the above agents were calculated as described [24].

Other demographic and clinical characteristics recorded at baseline included sex, age at PD onset, age at study enrollment, disease duration, dementia, Mini-Mental State Examination (MMSE) scores, history of psychosis, initial symptoms (tremor or not), presence of symptomatic hypotension, use of non-steroidal anti-inflammatory drugs, vascular risk factors including current smoker and comorbid diseases (diabetes mellitus, hypertension, and hyperlipidemia), and history of cardiovascular diseases and cerebral infarctions. Dementia was diagnosed based on the criteria of the Diagnostic and Statistical Manual of Mental Disorders (DSM-IV-TR) [25]. Symptomatic orthostatic hypotension was diagnosed according to UPDRS-IV. History of psychosis, including visual and auditory hallucinations and delusions, was obtained. These variables were assessed retrospectively by one investigator (AU) based on information in the patients’ medical records. UPDRS-III scores and concurrent medications were recorded at a median (IQR) of six (4–11) times, at median (IQR) intervals of 83 (48–144) days. CRP concentrations were measured at a median (IQR) of four (2–7) times, at median (IQR) intervals of 110 (59–213) days.

### Chronological stability of baseline CRP

To investigate the stability of baseline CRP concentrations, the follow-up period was subdivided into two periods, Days 1–180 and 181–360, and the average CRP concentrations in each were calculated. Patients were grouped into three tertiles by baseline plasma CRP concentration.

### Definition of study enrollment, observation period, and censoring

The date of study enrollment was defined as the date UPDRS-III score was first assessed; blood samples were screened for CRP measurements within one month [median (IQR) interval: 2 (0–11) days]. Patients were followed-up until Day 900, or October 31, 2014; patients lost to follow-up and those who experienced outcomes such as including stroke, brain surgery, fracture, or other types of surgery were censored at that time, because these conditions could have interfered with UPDRS-III scores. Patients were also censored at the time of any infection or acute inflammations requiring medical treatment.

### Variables

**Fig 1:**
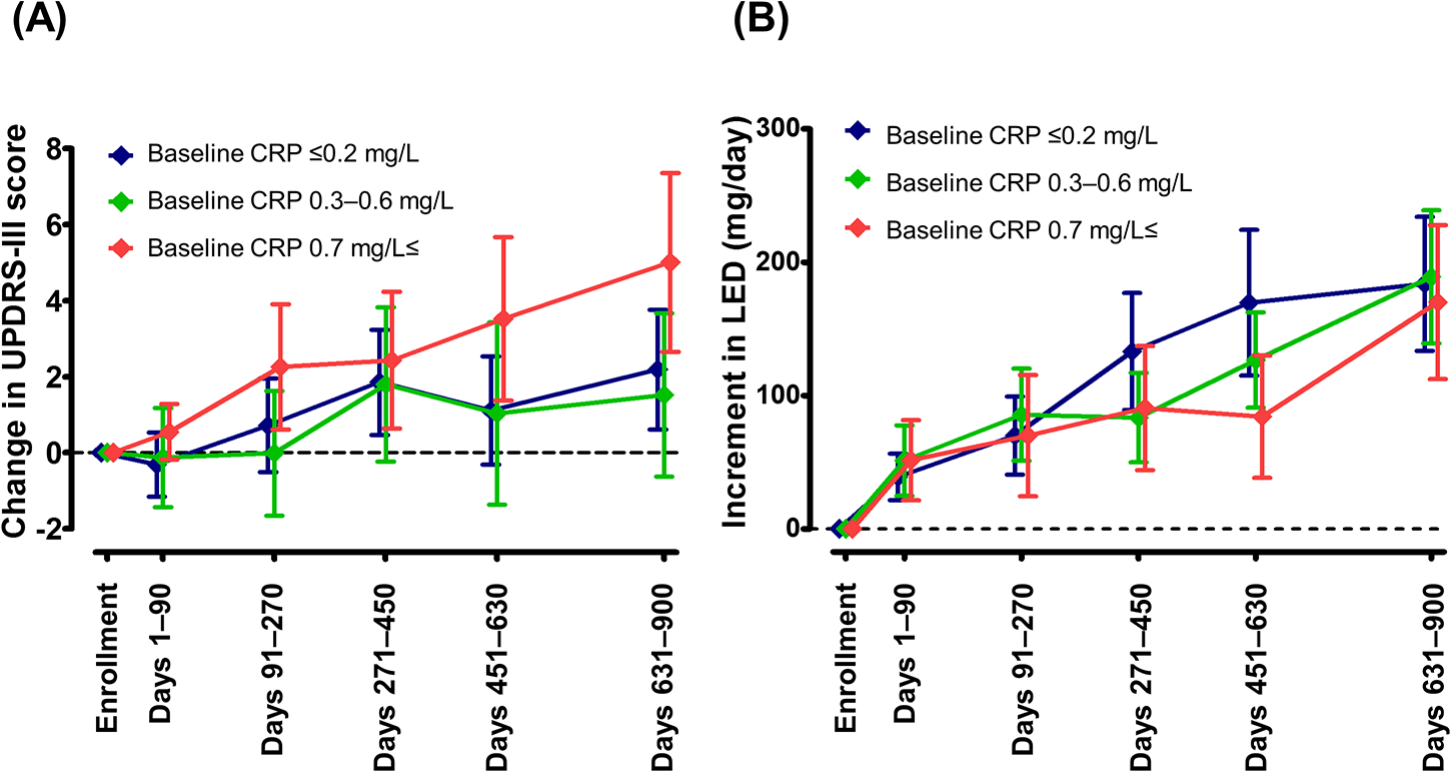
Changes in Unified Parkinson’s Disease Rating Scale Part III (UPDRS-III) scores (A) and L-dopa equivalent dose (LED) (B) by tertile of baseline C-reactive protein (CRP) concentrations. UPDRS-III score and LED (mg/day) were measured at the study enrollment and during the follow-up period. Subjects were sorted by baseline CRP concentration [≤0.2 mg/L (blue), 0.3–0.6 mg/L (green), and ≥0.7 mg/L (red)]. Symbols, and upper and lower error bars represent mean and 95% CI, respectively.

The observation period was divided into six sections (enrollment at Day 0, and follow-up periods at Days 1–90, 91–270, 271–450, 451–630, and 631–900). The average changes in UPDRS-III scores and concurrent LEDs at each follow-up period were calculated. Preliminary graphic analysis of changes in UPDRS-III scores indicated that these scores increased to a similar extent in the lowest (≤0.2 mg/L) and middle (0.3–0.6 mg/L) tertiles of baseline CRP concentrations, but not in the highest tertile (≥0.7 mg/L) (**Fig 1**); therefore, baseline CRP concentrations (<0.7 mg/L and ≥0.7 mg/L) were treated as ordinal variables. Each other variable was treated as follows: age at PD onset, age at study enrollment, disease duration, UPDRS-III scores, MMSE scores, and LED as scale variables; modified H-Y stage (1–2 and 2.5–4) as an ordinal variable; sex, dementia, history of psychosis, initial symptoms, presence of symptomatic orthostatic hypotension, use of non-steroidal anti-inflammatory drugs, smoking, diabetes mellitus, hypertension, hyperlipidemia, and history of cardiovascular diseases and cerebral infarctions as dichotomous nominal variables.

### Study size

In a previous study [26], the mean annual increase in UPDRS-III scores was 3.3 points. This study hypothesized that the mean (SD) difference in UPDRS-III scores between the bottom two-thirds and the top one-third of subjects sorted by baseline CRP concentrations would be 1.0 (2.0) point. Assuming a power of 90% and *P*<0.05, the sample size was estimated to be 192 patients.

### Statistical Analysis

Scale variables were compared between patients with in the top tertile and the bottom two tertiles of CRP concentrations using the Mann–Whitney U test, because of their non-Gaussian distribution. Ordinal and nominal variables were statistically analyzed using Fisher’s exact tests. The association between baseline CRP and change in UPDRS-III scores during the entire follow-up period (Analysis I) was analyzed by a generalized estimation equation with an exchangeable correlation structure [27]. First, to investigate the multicollinearity between scale variables, scattered plots of the correlation matrix were obtained (**S1 Fig**), showing multicollinearity between age at PD onset and actual age (Spearman’s rank correlation coefficient = 0.87, *P*<0.001). The repeatedly measured outcomes of changes in UPDRS-III score were clustered by study participant. Missing values of changes in the UPDRS-III score were excluded from the statistical models. To estimate its effect on motor prognosis, the regression coefficient of baseline CRP was calculated, adjusted for difference in LED (Model I), and additionally adjusted for other baseline characteristics, including sex, age at study enrollment, baseline UPDRS-III score, and dementia (Model II). Finally, the association between baseline CRP and change in UPDRS-III score during the final follow-up period (Days 631–900, Analysis II) was analyzed using generalized linear models (Models I and II) [28]. All statistical analyses were performed using GraphPad Prism for Windows ver. 5.0 (GraphPad Software, San Diego, CA, USA, http://www.graphpad.com), and SPSS 22.0 software (PASW statistics, http://www.spss.com/). *P* values less than 0.05 were considered statistically significant.

## Results

### Characteristics of study subjects

**Fig 2:**
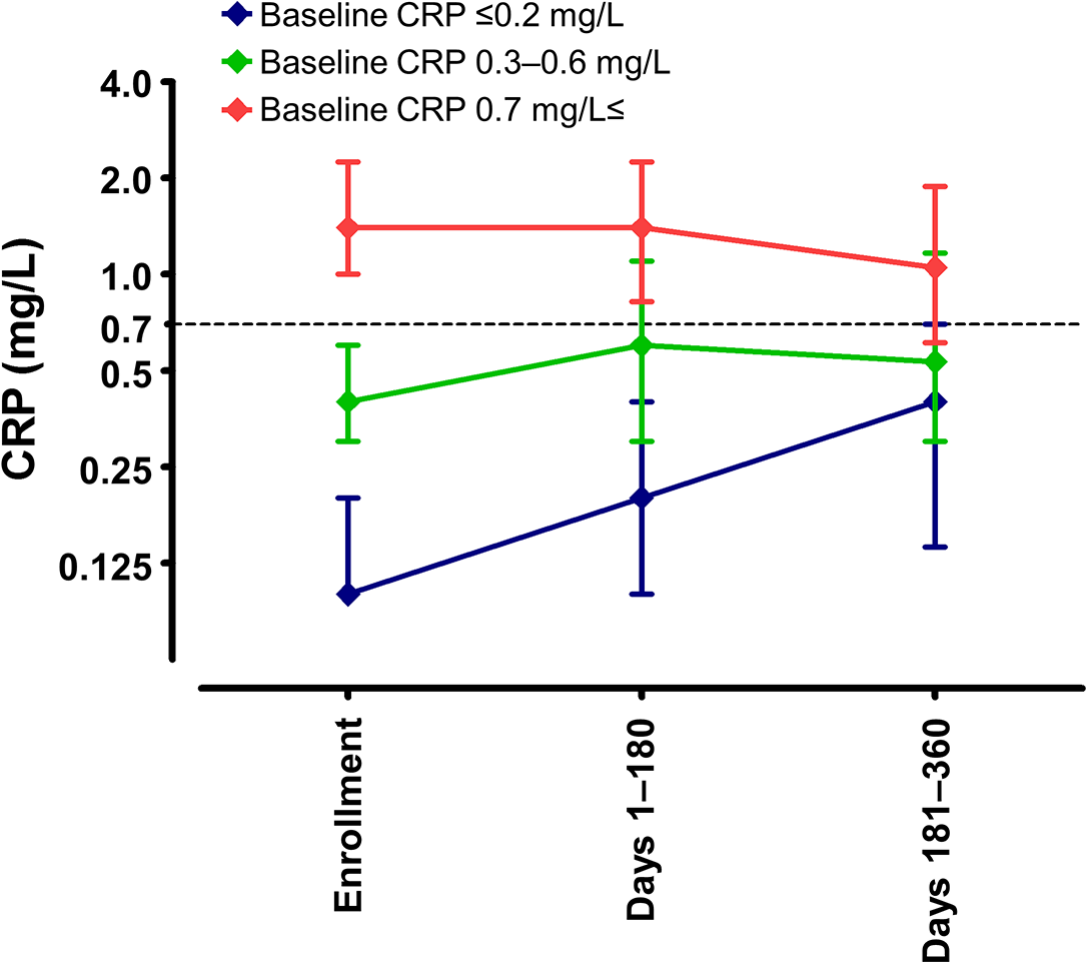
C-reactive protein (CRP) concentrations over time in Parkinson disease (PD) patients sorted by tertile of baseline CRP concentrations. CRP levels were measured at study enrollment and divided into tertiles [≤0.2 mg/L (blue), 0.3–0.6 mg/L (green), and ≥0.7 mg/L (red)], with CRP concentrations over time measured in each tertile. Symbols and upper and lower error bars represent median and 75th and 25th percentiles, respectively.

Of the 406 patients with PD, 31 were excluded, 3, 10, 9, 6, and 3 of whom developed dementia within 1 year of the onset of parkinsonism, and had history of DBS surgery, cancer, autoimmune diseases, and infection within one month prior to enrollment, respectively. The remaining 375 PD subjects (mean age, 69.3 years; mean PD duration, 6.6 years) were followed-up for a mean of 590 days (**S2 Fig**) This dataset has been registered in the Dryad Digital Repository (doi:10.5061/dryad.2bb46). Periodic measurements showed that changes in CRP concentrations were almost stable, including in the top CRP tertile (**Fig 2**). Baseline subject characteristics are shown in **Table 1**. Patients in the top CRP tertile were significantly older (*P* = 0.006), had a higher modified H-Y stage (*P* = 0.009), and had a lower MMSE score (*P* = 0.013) than patients in the lower two tertiles. Hypertension (*P* = 0.018) and hyperlipidemia (*P* = 0.025) were significantly more common in the top CRP tertile than in the bottom two tertiles. There were no other significant differences in baseline characteristics between the top and lower two tertiles of CRP concentrations.

**Table 1:** Baseline characteristics in patients sorted by tertiles of baseline C-reactive protein (CRP) concentration.

	Baseline CRP		
	bottom two thirds	top third	
	<0.7 mg/L	0.7 mg/L≤	*P*
N	247	128	
Sex (male), n [%]	98 [39.7]	58 [45.3]	0.321
Age of PD onset (years), median [IQR]	63.0 [56.0–69.0]	64.5 [58.0–70.0]	0.100
Age at study enrollment (years), median [IQR]	69.0 [64.0–74.0]	72.0 [66.0–77.0]	0.006
Disease duration (years), median [IQR]	5.0 [3.0–9.0]	6.0 [4.0–9.0]	0.057
Modified H-Y stage, n [%]			
1–2	85 [34.4]	27 [21.1]	0.009
2.5–4	162 [65.6]	101 [78.9]	
UPDRS-III score, median [IQR]	19.0 [13.0–25.0]	20.0 [14.0–26.0]	0.304
Dementia, n [%]	49 [19.8]	36 [28.1]	0.090
MMSE score, median [IQR], n	28.0 [25.0–30.0], 223	27.0 [24.0–29.0], 113	0.013
History of psychosis, n [%]	83 [33.6]	48 [37.5]	0.494
Initial symptoms, non-tremor, n [%]	127 [51.6], 246	61 [47.7]	0.514
Symptomatic orthostatic hypotension, n [%]	66 [26.7]	34 [26.6]	1.000
LED (mg/day), median [IQR]	450.0 [300.0–682.3]	536.0 [353.1–700.0]	0.230
Use of non-steroidal anti-inflammatory drugs, n [%]	34 [13.8]	24 [18.8]	0.229
Current smoker, n [%], n	6 [2.9], 205	3 [2.8], 106	1.000
Diabetes mellitus, n [%]	22 [8.9]	12 [9.4]	0.852
Hypertension, n [%]	66 [26.7]	50 [39.1]	0.018
Hyperlipidemia, n [%]	54 [21.9]	42 [32.8]	0.025
History of cardiovascular diseases, n [%]	4 [1.6]	5 [3.9]	0.284
History of cerebral infarction, n [%]	4 [1.6]	2 [1.6]	1.000

### Longitudinal analysis of effect of plasma CRP on motor prognosis

#### Analysis I: effect during the entire follow-up period

A total of 1,050 clustering points was analyzed by a generalized estimation equation model for the entire follow-up period. A statistical model incorporating baseline CRP and change in LED showed that baseline CRP was significantly associated with changes in UPDRS-III score (*P* = 0.006), with a regression coefficient of 1.78 [95% confidence interval (CI) 0.51–3.06] (**Table 2**, Model I). After adjustment for baseline characteristics, including sex, age, baseline UPDRS-III score, and dementia, baseline CRP was found to be significantly associated with change in UPDRS-III score [regression coefficient = 1.41 (95% CI 0.21–2.61), *P* = 0.021] (**Table 2**, Model II).

**Table 2:** Multivariate models of effect of baseline C-reactive protein (CRP) on change in Unified Parkinson’s Disease Rating Scale Part III (UPDRS-III) score for the entire follow-up period, as estimated by a generalized estimation equation model.

Variable	B	95% CI	*P*
Model I			
Baseline CRP			
<0.7 mg/L (reference)	0		
0.7 mg/L≤	1.78	0.51–3.06	0.006
Increment in LED			
Per 100 mg/day increase	-0.23	-0.49–0.03	0.080
Model II			
Baseline CRP			
<0.7 mg/L (reference)	0		
0.7 mg/L≤	1.41	0.21–2.61	0.021
Sex			
Female (reference)	0		
Male	1.40	0.30–2.51	0.013
Age			
Per five years increase	0.84	0.53–1.16	<0.001
Baseline UPDRS-III score			
Per five points increase	-1.37	-1.70–-1.04	<0.001
Dementia			
No (reference)	0		
Yes	0.85	-0.67–2.37	0.272
Increment in LED			
Per 100 mg/day increase	-0.19	-0.44–0.06	0.132

#### Analysis II: effect for the final period

To assess the effect of CRP on motor prognosis for the final follow-up period (Days 631–900), a regression coefficient of CRP was calculated using a generalized linear model. Of the 375 participants, 175 were excluded because of their censoring (**S1 Table**), and the remaining 200 were analyzed. A generalized linear model showed that baseline CRP was significantly associated with change in UPDRS-III score, both for Model I, adjusted for change in LED [regression coefficient = 2.99 (95% CI 0.43–5.55), *P* = 0.022] and for Model II, adjusted for change in LED and baseline characteristics [regression coefficient = 2.62 (95% CI 0.25–4.98), *P* = 0.030] (**Table 3**).

**Table 3:** Multivariate models of effect of baseline CRP on change in UPDRS-III score for the final follow-up period (Days 631–900), as estimated by a generalized linear model.

Variable	B	95% CI	*P*
Model I			
Baseline CRP			
<0.7 mg/L (reference)	0		
0.7 mg/L≤	2.99	0.43–5.55	0.022
Increment in LED			
Per 100 mg/day increase	-0.66	-1.18–-0.14	0.013
Model II			
Baseline CRP			
<0.7 mg/L (reference)	0		
0.7 mg/L≤	2.62	0.25–4.98	0.030
Sex			
Female (reference)	0		
Male	1.21	-0.90–3.31	0.261
Age			
Per five years increase	1.28	0.75–1.81	<0.001
Baseline UPDRS-III score			
Per five points increase	-1.62	-2.19–-1.06	<0.001
Dementia			
No (reference)	0		
Yes	3.01	-0.08–6.10	0.056
Increment in LED			
Per 100 mg/day increase	-0.42	-0.86–0.02	0.061

### Comparison of CRP concentrations in PD patients and controls

Plasma CRP was also measured in 65 controls [27 males (41.5%)] of median (IQR) age, 63 (54.5–69.5) years. Median (IQR) CRP concentrations in these individuals were 0.60 (0.30–1.25) mg/L. The distribution of CRP levels differed slightly but significantly between PD patients and controls (Mann–Whitney U test, *P* = 0.003) (**S3 Fig**).

## Discussion

To our knowledge, all previous studies assessing CRP and motor symptoms in PD patients were cross-sectional in design [29,30], with none evaluating the long-time effect of CRP on motor prognosis. One study reported a positive correlation between UPDRS-III scores and CRP concentrations [29], while another did not [30]. In this study, there was no significant difference in baseline UPDRS-III scores between patients in the top tertile of CRP concentration and those in the bottom two tertiles. Moreover, at baseline, there was no significant correlation between UPDRS-III scores and CRP levels (Spearman’s rank correlation coefficient = 0.071, *P* = 0.169). Although higher CRP levels might be a consequence, not a cause, of more advanced stage disease, the association between CRP and motor prognosis was analyzed longitudinally, not cross-sectionally. The two parameters showed a statistically significant correlation, even after adjusting for change in LED and baseline characteristics. A community-based study [31] showed that plasma CRP concentration in individuals free of inflammations was highly associated with CRP gene polymorphisms. CRP levels were distributed similarly in PD patients and controls, but were slightly higher in controls, consistent with our previous findings [32]. Although it has not been determined whether subclinical systemic inflammation is associated with neuroinflammation, individuals with subclinically high CRP levels were found to be at increased risk of developing Alzheimer’s disease [10]. Several studies have suggested that levels of inflammatory cytokines are elevated in patients with PD [33–35], and subclinically high plasma concentrations of interleukin-6 have been associated with the development of PD [36]. Elevated plasma CRP concentrations before the onset of parkinsonism may therefore have an enduring effect on motor prognosis.

In Japanese populations, PD occurs more frequently in females than in males [37,38], whereas, in other populations, PD occurs more frequently in males [39,40]. The motor prognosis of PD was also poorer in older and in male patients, consistent with previous findings [41]. Baseline UPDRS-III score showed a negative association with motor prognosis. UPDRS-III score is sensitive to change, especially in early stages of PD [21], but reaches a ceiling during advanced stage PD. This score was not significantly associated with dementia, unlike in a previous study [41]; however, three patients who developed dementia within 1 year of the onset of parkinsonism were excluded. Comparing with the eligible patients, the subjects who were excluded due to development of dementia within 1 year were significantly greater in UPDRS-III score, and had similar CRP levels, while they had elderly onset and took lower LEDs (**data not shown**).

During the fourth follow-up period (Days 451–630), the change in UPDRS-III score was greater (*P* = 0.045), but the change in LED was smaller (*P* = 0.002), in patients in the top than in the bottom two tertiles of baseline CRP. The prevalence of psychosis did not differ significantly between the top and bottom two tertiles of baseline CRP, but was significantly higher in the top tertile during the fourth follow-up period (**S2 Table**), suggesting that the increment of dopamine replacement therapy was limited due to comorbid psychosis in the top tertile. UPDRS-III scores were measured during the “on period”. These scores can be modified by anti-Parkinsonian agents. Although that is a limitation of this study, change in UPDRS-III score is a key clinical parameter in the treatment of PD.

In summary, this study showed that the subclinical elevation of plasma CRP has a strong impact on motor prognosis in PD, suggesting that subclinical systemic inflammation could affect neurodegeneration in PD.

## Supporting Information

S1 Fig Matrix scatter plots of predictable variables. Scale variables (age, age at onset, disease duration, and Unified Parkinson’s Disease Rating Scale Part III scores) were plotted to check their multicollinearity. Multicollinearity was observed between age and age at onset.
(PPTX)

S2 Fig Flow diagram of the study enrollment process. PD, Parkinson disease; H-Y, Hoehn and Yahr; DBS, deep brain stimulation. Causes of censoring for analysis during the final period (Days 631–900) are shown in **S1 Table**.
(PPTX)

S3 Fig Histogram of log_2_C-reactive protein (CRP) concentrations in Parkinson disease (PD) patients (green) and controls (blue). Log_2_CRP was distributed as a bell-shaped curve. The distribution of CRP concentrations was very similar to, but slightly higher in, controls than in PD patients (Mann–Whitney U test, *P* = 0.003).
(PPTX)

S1 Table Causes of censoring for analysis during the final period (Days 631–900). (DOC)

S2 Table Patient characteristics at baseline and during the fourth period (Days 451–630) by tertiles of baseline C-reactive protein (CRP). (DOC)
