# Supplementary figures and images for: Baseline Plasma C-Reactive Protein Concentrations and Motor Prognosis in Parkinson Disease
Source: PLoS One. 2015 Aug 26;10(8):e0136722. doi: 10.1371/journal.pone.0136722 (PMC4550234; doi:10.1371/journal.pone.0136722)

## Slide 1
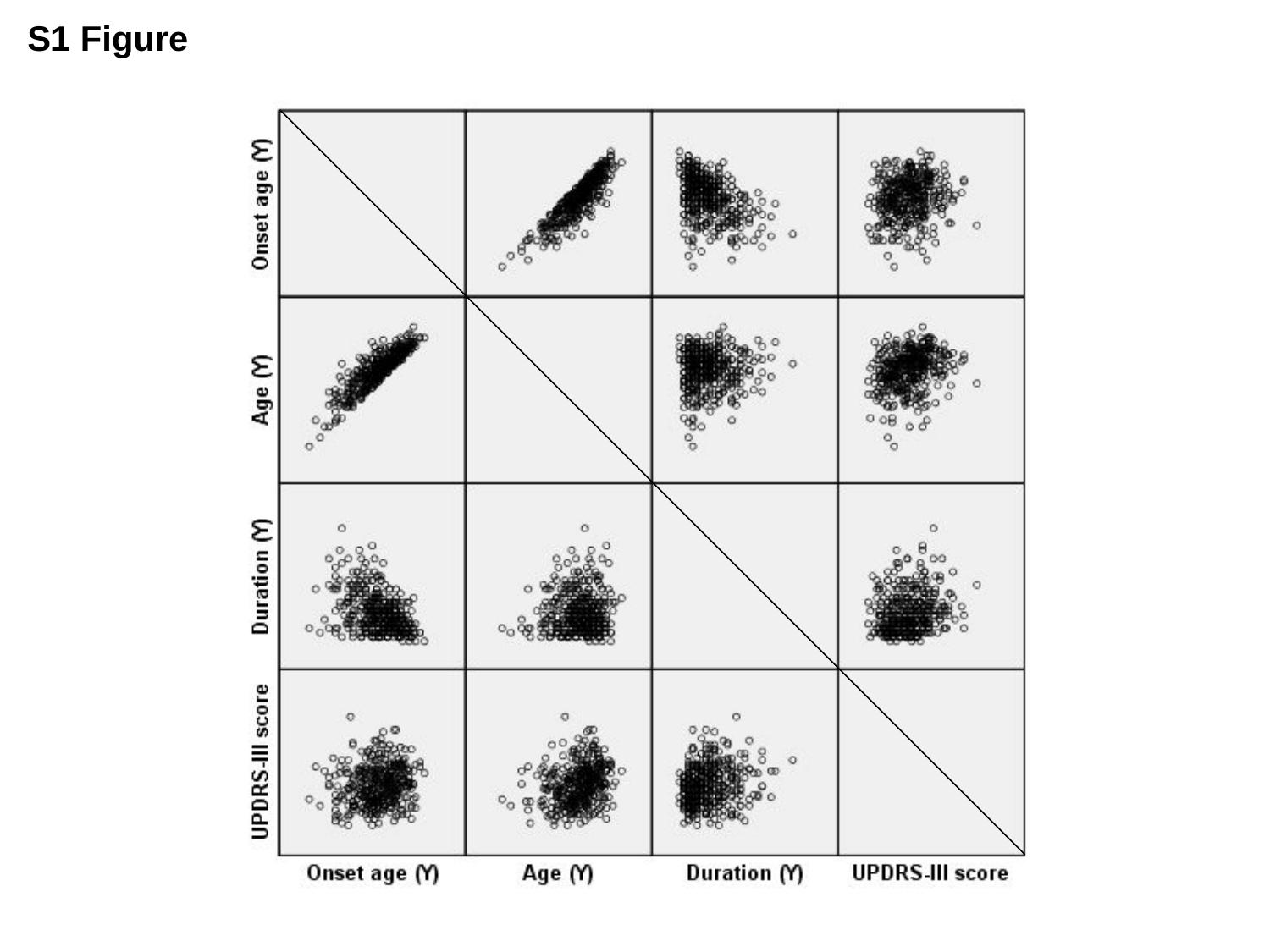

S1 Figure

Supplement: S1 Fig — Scale variables (age, age at onset, disease duration, and Unified Parkinson’s Disease Rating Scale Part III scores) were plotted to check their multicollinearity. Multicollinearity was observed between age and age at onset. (PPTX) [file pone.0136722.s001.pptx]

## Slide 1
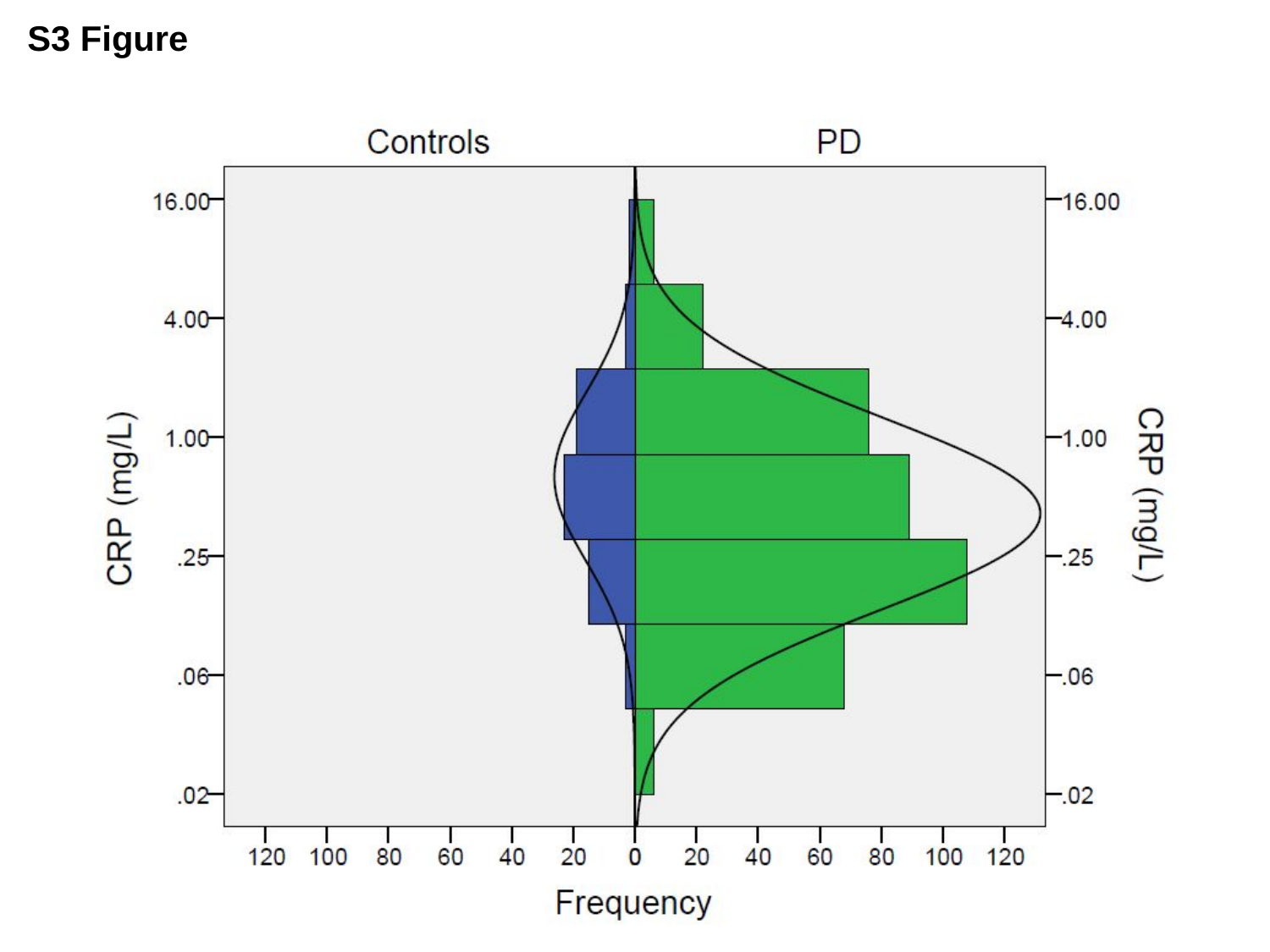

S3 Figure

Supplement: S3 Fig — Log2CRP was distributed as a bell-shaped curve. The distribution of CRP concentrations was very similar to, but slightly higher in, controls than in PD patients (Mann–Whitney U test, P = 0.003). (PPTX) [file pone.0136722.s003.pptx]
