# Supplementary material for: Baseline Plasma C-Reactive Protein Concentrations and Motor Prognosis in Parkinson Disease
Source: PLoS One. 2015 Aug 26;10(8):e0136722. doi: 10.1371/journal.pone.0136722 (PMC4550234; doi:10.1371/journal.pone.0136722)
Supplement: S2 Fig — PD, Parkinson disease; H-Y, Hoehn and Yahr; DBS, deep brain stimulation. Causes of censoring for analysis during the final period (Days 631–900) are shown in S1 Table. (PPTX) [file pone.0136722.s002.pptx]

## Slide 1
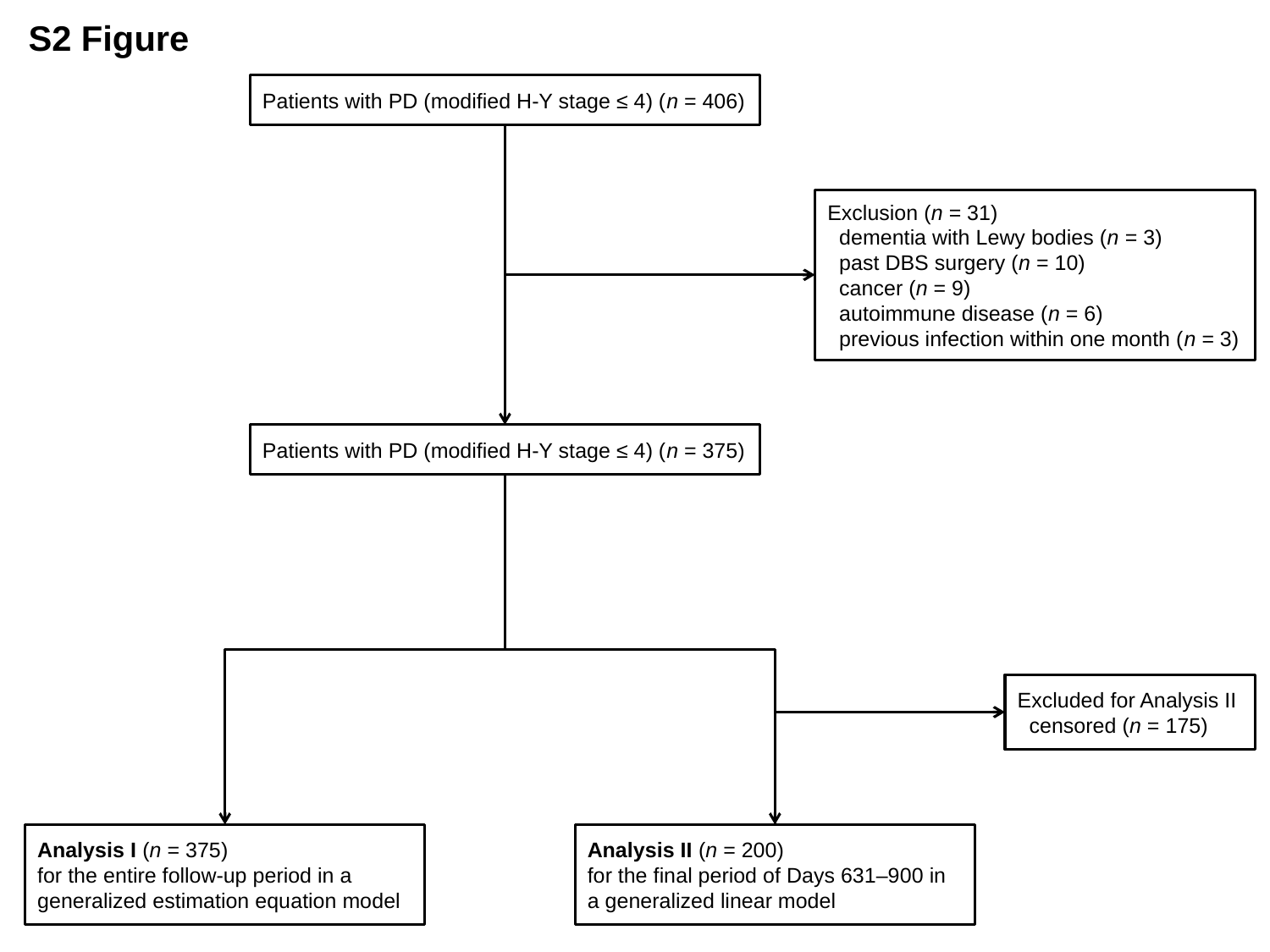

S2 Figure
Patients with PD (modified H-Y stage ≤ 4) (n = 406)
Exclusion (n = 31)
 dementia with Lewy bodies (n = 3)
 past DBS surgery (n = 10)
 cancer (n = 9)
 autoimmune disease (n = 6)
 previous infection within one month (n = 3)
Patients with PD (modified H-Y stage ≤ 4) (n = 375)
Excluded for Analysis II
 censored (n = 175)
Analysis I (n = 375)
for the entire follow-up period in a generalized estimation equation model
Analysis II (n = 200)
for the final period of Days 631–900 in a generalized linear model
