# Supplementary material for: Baseline Plasma C-Reactive Protein Concentrations and Motor Prognosis in Parkinson Disease
Source: PLoS One. 2015 Aug 26;10(8):e0136722. doi: 10.1371/journal.pone.0136722 (PMC4550234; doi:10.1371/journal.pone.0136722)
Supplement: S1 Table — (DOC) [file pone.0136722.s004.doc]

**S1 Table. Causes of censoring for analysis during the final period (Days 631–900).**

|  | N | % |
| --- | --- | --- |
| Lost | 137 | 78.3 |
| Alternative outcomes |  |  |
| Infections ^a^ | 16 | 9.1 |
| Fractures ^b^ | 12 | 6.9 |
| Inflammation ^c^ | 6 | 3.4 |
| Sudden death | 2 | 1.1 |
| Cerebral infarction | 1 | 0.6 |
| Surgery (bunion) | 1 | 0.6 |
| Total | 175 | 100.0 |

^a^ pneumonia (n=10), urinary tract infection (n=4), colitis (n=1), and cholecystitis (n=1)

^b^ femoral bone (n=3), vertebrae (n=4), pelvic bone (n=2), jawbone (n=1), rib (n=1), and fibula (n=1)

^c^ malignant syndrome (n=2), acute myocardial infarction (n=2), drug allergy (n=1), and pemphigoid (n=1)
