# Supplementary material for: Baseline Plasma C-Reactive Protein Concentrations and Motor Prognosis in Parkinson Disease
Source: PLoS One. 2015 Aug 26;10(8):e0136722. doi: 10.1371/journal.pone.0136722 (PMC4550234; doi:10.1371/journal.pone.0136722)
Supplement: S2 Table — (DOC) [file pone.0136722.s005.doc]

**S2 Table. Patient characteristics at baseline and during the fourth period (Days 451–630) by tertiles of baseline C-reactive protein (CRP).**

|  | Baseline CRP |  |  |
| --- | --- | --- | --- |
|  | bottom two thirds | top third |  |
|  | <0.7 mg/L | 0.7 mg/L≤ | *P* |
| N | 124 | 63 |  |
| Sex (male), n [%] | 56 [45.2] | 34 [54.0] | 0.281 |
| Age of PD onset (years), median [IQR] | 63.0 [55.0–68.0] | 64.0 [59.0–70.0] | 0.108 |
| Age at study enrollment (years), median [IQR] | 69.0 [62.0–74.0] | 72.0 [65.0–77.0] | 0.016 |
| Disease duration (years), median [IQR] | 5.0 [3.0–8.8] | 6.0 [4.0­–9.0] | 0.215 |
| UPDRS-III score, median [IQR] | 19.0 [13.0–25.0] | 19.0 [11.0–28.0] | 0.977 |
| LED (mg/day), median [IQR] |  |  |  |
| At study enrollment | 450.0 [300.0–650.0] | 550.0 [370.0–700.0] | 0.092 |
| At Days 451–630 | 599.3 [412.5–799.6] | 625.0 [500.0–750.0] | 0.656 |
| History of psychosis, n [%] |  |  |  |
| At study enrollment | 35 [28.2] | 20 [31.7] | 0.615 |
| At Days 451–630 | 44 [35.5] | 34 [54.0] | 0.019 |
